# Dried urine spot method for detection of *Schistosoma mansoni* circulating cathodic antigen in resource-limited settings: a proof of concept study

**DOI:** 10.3389/fimmu.2023.1216710

**Published:** 2023-09-11

**Authors:** Abdallah Zacharia, Twilumba Makene, Clemence Kinabo, George Ogweno, Faraja Lyamuya, Billy Ngasala

**Affiliations:** ^1^ Department of Parasitology and Medical Entomology, Muhimbili University of Health and Allied Sciences, Dar es Salaam, Tanzania; ^2^ National Institute for Medical Research, Mwanza, Tanzania; ^3^ Neglected Tropical Diseases Control Program, Ministry of Health, Dodoma, Tanzania

**Keywords:** dried matrix spot, dried urine spot, extraction, elution, Schistosoma mansoni, circulating cathodic antigens, filter paper

## Abstract

**Background:**

Among the challenges in schistosomiasis surveillance and mapping surveys is the lack of a sensitive diagnostic method especially in low transmission setting. Currently, the WHO recommends the use point-of-care circulating cathodic antigen (Schisto POC-CCA) tests for surveillance and mapping of intestinal schistosomiasis. However, Schisto POC-CCA test has its drawbacks, one of which is the timely availability of test kits. One approach to overcoming this challenge is to develop a low-cost sampling method that allows for the collection and transport of urine specimens even in resource-limited settings.

**Objective:**

To develop a simple and efficient method for the collection and detection of *Schistosoma mansoni* (*S. mansoni*) CCA using urine spotted onto filter paper.

**Methodology:**

To develop a dried urine spot (DUS) method, various dried matrix extraction parameters were tested and optimized using predesigned steps. The parameters include the size of filter paper (determined by the number of punches), volume of solvents, and type of solvent. Moreover, we optimized the incubation conditions (time and temperature). Urine and stool specimens to conduct the experiments were collected from volunteer fishermen in Mwanza and this project staff. Data were entered into the Microsoft Excel spreadsheet and IBM Statistical Package for the Social Sciences, version 20 for analysis.

**Results:**

The optimal results were obtained when the procedure was run under the following conditions: Five punches of filter paper containing DUS were dissolved in 150 µl of distilled water and incubated at room temperature for 24 hours in an Eppendorf tube. More than 93% of the assays performed under these conditions produced results that were either comparable to or significantly better than the standard method.

**Conclusion:**

This study demonstrates the feasibility of collecting urine specimen (DUS) using filter paper and detecting *Schistosoma* CCA from DUS specimen using the Schisto POC-CCA cassette test.

## 1 Introduction

Schistosomiasis is a parasitic disease caused by a trematode that belongs to the genus *Schistosoma*. Globally, approximately 240 million people are infected (about 90% live in sub-Saharan Africa), causing about 70 million disability-adjusted life years lost (1, 2). Widely used schistosomiasis diagnostic techniques include the Kato-Katz thick-smear and urine filtration for intestinal schistosomiasis and urogenital schistosomiasis respectively (3). But these methods have several pitfalls. First, they are time-consuming and require skilled laboratory technologists. In addition, they need good-quality microscopes and light sources (electricity), which makes them difficult to use in resource-limited areas (4). Moreover, the procedures are less sensitive when used in low-endemic settings (3). Hence, a highly sensitive and rapid diagnostic test is of paramount importance for successful schistosomiasis surveillance and mapping during the elimination stage (5).

Recently, there has been the development of more sensitive diagnostic tests based on the detection of *Schistosoma* antigens. The most commonly developed tests utilize the circulating antigens produced by adult worms. These antigens include circulating cathodic antigens (CCA) and circulating anodic antigens (CAA) (6). The CCA and CAA detection tests are useful for control programs because they are more sensitive than microscopy and hence provide more accurate information regarding the prevalence of *Schistosoma* infections (7, 8). Currently, the WHO has recommended the use of point-of-care CCA cassettes (Schisto POC-CCA) for mapping the prevalence and surveillance of *Schistosoma mansoni* by control programs (9). Despite its high sensitivity and specificity, the CAA test could not be used in the field as it involves steps that require laboratory equipment (8, 10). But also, the Schisto POC-CCA cassettes are commercially available from a single manufacture (Rapid Medical Diagnostics; Cape Town, South Africa) (11). The presence of a single manufacture impedes the timely availability of the tests (10). Additionally, schistosomiasis control programs in most endemic countries rely on the support of their implementing partners (donors), and the implementation of their activities are heavily reliant on the availability of the support from those partners (12). Furthermore, there are times when, as was the case during the COVID-19 pandemic, industrial production is reduced and transportation of materials within and across continents is difficult. Therefore, if there is a delay in receiving Schisto POC-CCA cassettes from the supplier or the support from the partners to purchase the cassettes, the program may require the use of cost-effective sample collection and storage methods that could be used even in a resource-limited setting where there is a high burden of schistosomiasis, especially when time for data (specimen) collection is a critical aspect of the particular work. This demand for the development of a simple and cost-effective sampling technique that will be used during sample collection, transportation, and storage in resource-limited settings.

*Schistosoma* circulating antigens are stable and detectable in urine, blood, and serum specimens. Urine specimens have several advantages over blood and serum. First, a sample of urine can stay for several days without a cold chain. Also, urine collection is noninvasive and doesn’t require trained medical personnel. Moreover, a large volume can easily be collected (11). However, liquid urine has an increased risk of infection (leakage). Furthermore, in resource-limited settings, the liquid urine is difficult to transport (heavy load of urine containers) and store (large space requirement). A dried urine spot (DUS) using filter papers is considered simple and cost-effective compared to liquid urine. DUS can be easily prepared in a field setting with less skills, easily transported, and stored with a minimum risk of infection (13). Studies have reported the use of filter papers for the detection of *Schistosoma* eggs and nucleic acid amplification (13, 14). However, no study was conducted to determine the use of DUS for the detection of *Schistosoma* antigens.

We aimed to develop a simple, less expensive and efficient method for collecting and detecting *Schistosoma mansoni* (*S. mansoni*) CCA using filter paper-based DUS in resource-limited settings. The method was developed by assessing and optimizing the performance of various dried matrix spot (DMS) parameters in order to find those that best fit for the filter paper-based DUS for maximum detection of *Schistosoma* CCA using the Schisto POC-CCA cassette test.

## 2 Methods

### 2.1 Type of the study

This laboratory-based experimental study was conducted to devise a method for collecting DUS on filter paper and extracting *S. mansoni* CCA from the collected DUS. The experimental study was carried out to test the feasibility of the predesigned steps for filter paper-based DUS method, as well as optimization of the materials and reagents to be used in the procedure and some parameters such as incubation temperature, solvent type and volume, and incubation time. The experiments were performed at parasitology laboratory of the National Institute for Medical Research (Mwanza Centre).

### 2.2 Development of dried urine spot method

#### 2.2.1 Sample collection

Fourteen volunteer fishermen aged 18 and older, as well as one volunteer study staff member with no history of exposure to *S. mansoni* risk environments, provided urine and stool specimens. Fishermen and volunteer staff member were given pre-labeled wide-mouthed urine and stool containers after providing informed consent for the collection of fresh urine and stool specimens. They were asked to collect approximately 30 mL of fresh urine and about 20 g of fresh stool specimens. Both urine and stool specimens were immediately transported to the National Institute for Medical Research (NIMR) Mwanza laboratory and processed using the Schisto POC-CCA cassettes (Rapid Medical Diagnostics, Cape Town, South Africa; batch number 220701075), and filtration technique (Shenzhen combined biotech co., Ltd (15), Guangdong, China) for direct urine tests, while stool samples were processed using the formal-ether sedimentation method and Kato-Katz techniques (Vestergaard Frandesen Group, Lausanne Switzerland) (16). The results of all procedures were reported as positive in case of the presence of *Schistosoma* eggs or CCA and negative in case of the absence of *Schistosoma* eggs or CCA. Furthermore, positive Schisto POC-CCA test results were further classified as trace, 1+, 2+, and 3+ according to the visibility of colour reaction of the particular test (17), whereas Kato-Katz results were further classified as light (1-99 EPG), moderate (100-399 EPG), and heavy (> 400 EPG) intensities (18). As with the direct urine test, the Schisto POC-CCA cassette procedure was used to conduct direct tests of the three solvents (normal saline, phosphate buffered saline, and distilled water) as part of quality control.

#### 2.2.2 Preparation of dried urine spots

Using a Pasteur pipette, urine was spotted on prelabeled 3x4-inch Whatman No. 3 (GE Healthcare UK Limited, Buckinghamshire, UK; Lot number 16988762) filter papers. To aid in the identification of the spots both immediately after application and after drying, urine was spotted on four printed circles. Five to 6 drops of urine were sufficient to saturate one printed circle. Four types of DUS were prepared using three positive urine specimens from fishermen with Kato-Katz (standard Schisto POC CCA) results: one light (2+), one moderate (3+), and one heavy (3+), as well as one urine sample from volunteer staff with negative Kato-Katz and Schisto POC-CCA results. The spotted filter papers were air dried for 24 hours in a fly-free box before being stored at room temperature in a small ziplock bag with a desiccant. The same procedure was used to prepare dried solvent spots using the three extraction solvents tested during the experiments (normal saline, phosphate buffered saline, and distilled water) as no template controls.

#### 2.2.3 Optimization of the number of punches and solvent volume

A series of trial-and-error experiments were conducted to determine the number of DUS punches that can be dissolved in a solvent and yield a sufficient volume (2 drops of Schisto POC-CCA pipette ≈ 100 µl) of eluate for Schisto POC-CCA cassette testing, as well as having strong visual (colour) resemblance with original urine sample. Briefly, using a single hole handheld punching machine (6 mm diameter), filter paper with DUS was punched into an Eppendorf tube (1.5 ml capacity) and a specific volume of solvent (phosphate buffered saline, distilled water or normal saline) was added. The Eppendorf tube was then incubated at room temperature for approximately 2 hours. Following incubation, a micropipette with a 100 µl marked tip was used to measure the volume of the eluate before visual (colour) observation. The visual observation was given a grade of weak if the elute just slightly resembled the original urine, moderate if it did so somewhat, and strong if it did so closely. The trial started with 3 punches into 120 µl of solvent and ended with 5 punches into 150 µl of solvent.

#### 2.2.4 Extraction of *Schistosoma mansoni* circulating cathodic antigen

**Figure 1:**
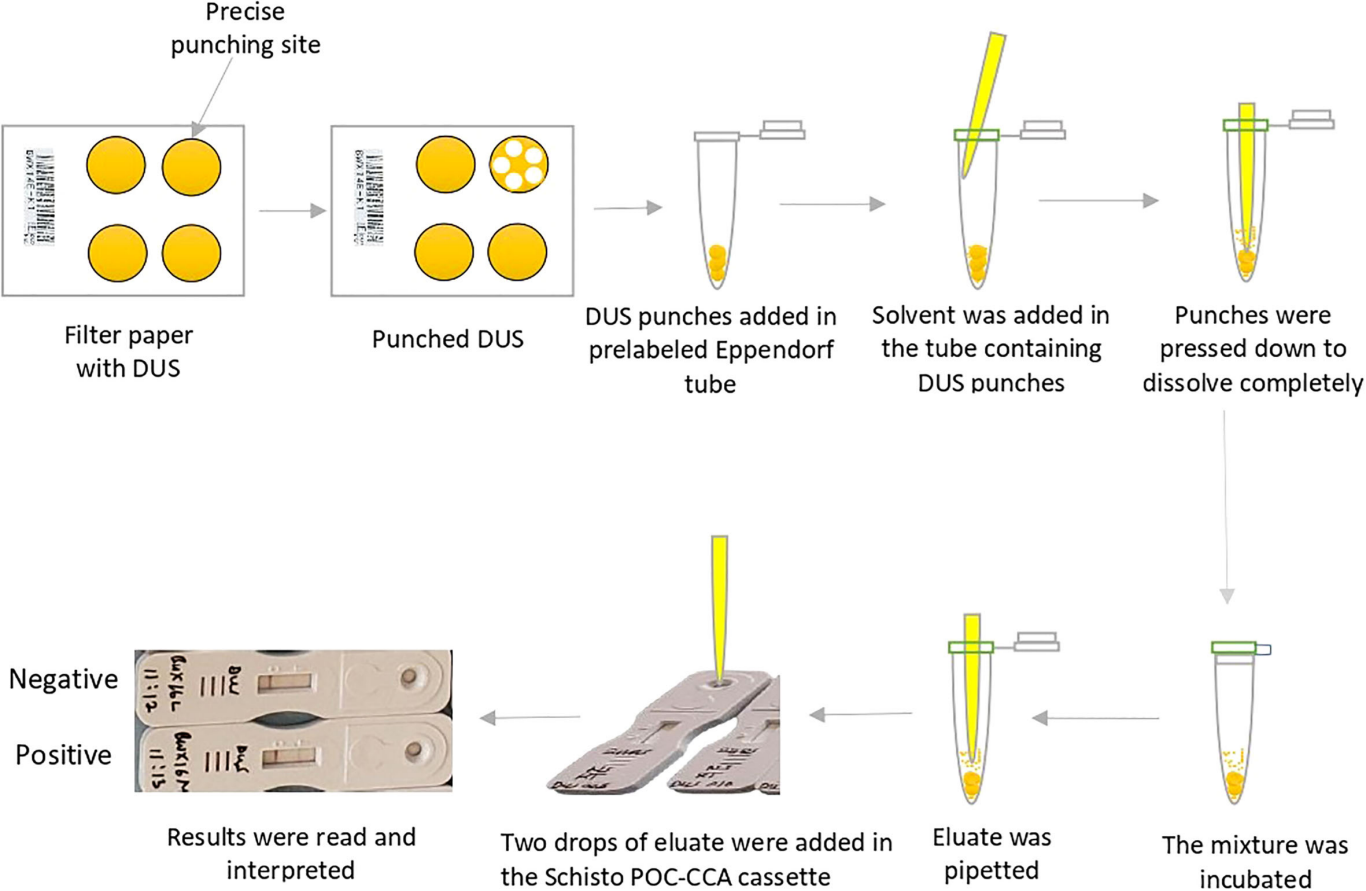
Step-by-step procedure for *Schistosoma mansoni* circulating cathodic antigens extraction from dried urine spot.

**Figure 2:**
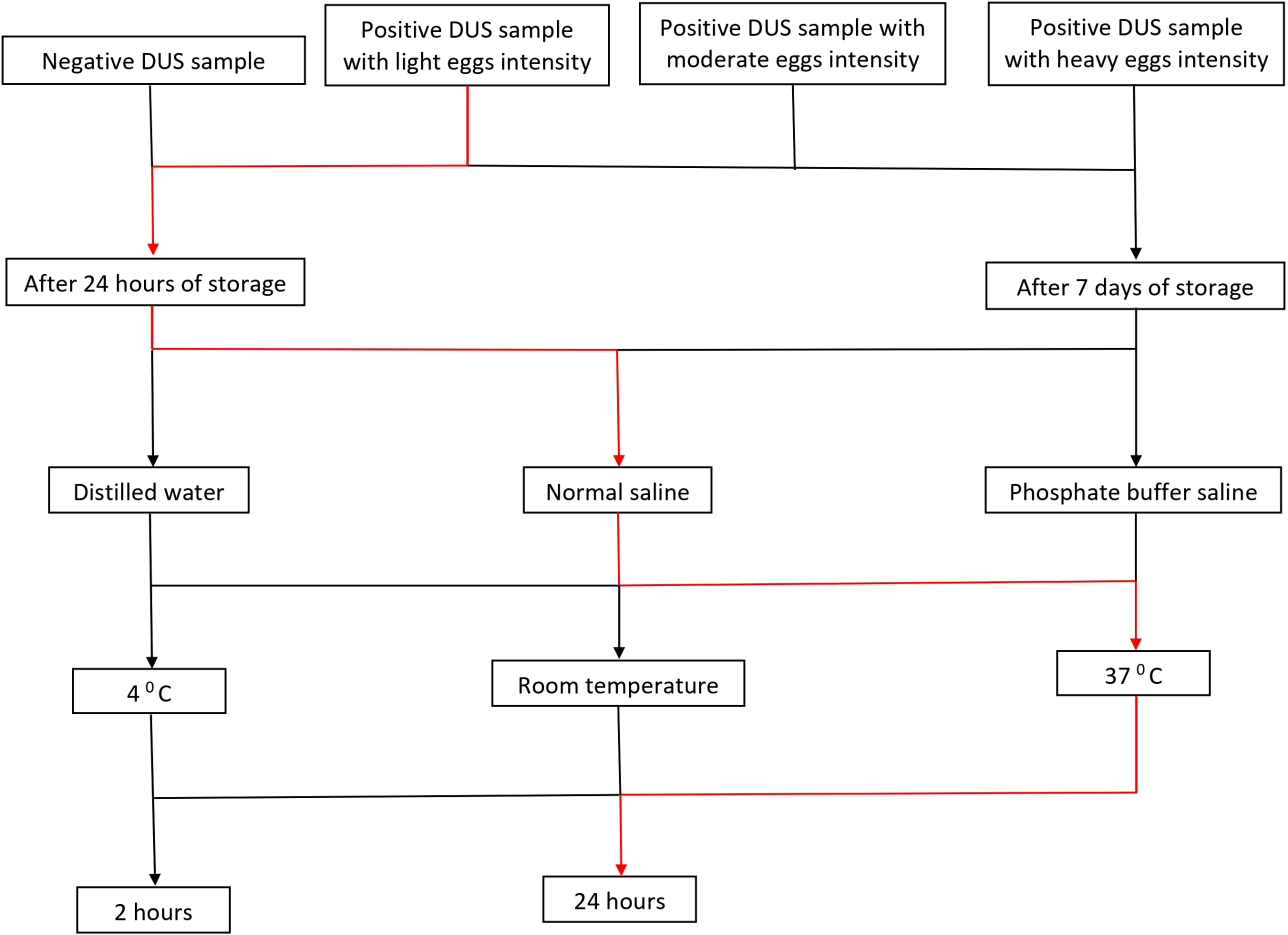
Flow chart illustrating the conditions that have been included in each experiment. Note: a red line depicts an assay experiment performed on the DUS sample with light intensity of infection that has been stored for 24 hours. The assay has been done using normal saline as extraction solvent, incubated at 37 ^0^C for 24 hours.

The maximum recovery of three different eluting solvents (phosphate buffered saline pH 9, distilled water, and normal saline) at different temperatures (4^0^ C, room temperature, and 37^0^ C) and time conditions (2 hours and 24 hours) were determined 24 hours and 7 days after storing DUS in ziplock bags at room temperature. **Figure 1** depicts the step-by-step procedure. In brief, the filter paper containing DUS as prepared in Section 2.2.2 was transferred from the storage area to the punching bench. Then, the precise location of the specimen spot on the filter paper was pinpointed. This was followed by punching 5 DUS disks (punches with 6 mm diameter each) in a prelabeled Eppendorf tube. The Eppendorf tube containing the 5 punches was then filled with one of the three solvents in the amount of 150 µl. The five DUS punches were pressed to the bottom of the tube with the pointed end of an unused micropipette tip to ensure that they were completely dissolved in the solvent. The tube was then incubated at one of three temperatures and one of the two incubation time. Therefore, 36 Eppendorf tubes were prepared on each of the four types of DUS specimens (18 Eppendorf tubes after 24 hours and the other 18 Eppendorf tubes 7 days after storage). Each one of the 18 Eppendorf tubes differed from the others in one of the following ways: the type of solvent it contained, the temperature at which it was incubated, or the time of incubation (**Figure 2**). As part of quality control, the same procedure was performed for dried solvent spots.

#### 2.2.5 Detection of *Schistosoma mansoni* circulating cathodic antigens

The *S. mansoni* CCA detection was carried out in accordance with the Schisto POC-CCA procedure manual, with minor modifications. To summarize, all assay materials including Eppendorf tubes containing the mixture of filter paper-based DUS punches and the extracting solvents were first brought to room temperature. To separate the eluate from the punches and allow the pipette to reach the bottom of the tube, the dissolved punches were pressed on one side of the Eppendorf tube with the pipette provided in the Schisto POC-CCA kit. The pipette was then removed from the tube, squeezed, and reinserted with the tip touching the bottom of the tube. By gently releasing the pipette, the eluate was allowed to fill up. Two drops of eluate (equivalent to 90-100 µl) were transferred into the circular well of the prelabeled Schisto POC-CCA test cassette by gently squeezing the pipette. The eluate was allowed to completely absorb into the specimen pad contained within the circular well. The time for reading the results was then marked on the test cassette. Twenty minutes after the sample was added to the circular well, the result was read and recorded in the results form as negative or positive (trace, 1+, 2+, or 3+) (15).

### 2.3 Data analysis

The collected data was entered into Microsoft Excel spreadsheet before being transferred to the Statistical Package for the Social Sciences Version 20 for analysis. The prevalence of *Schistosoma* infection among fishermen was calculated for each diagnostic technique. For each DUS extraction condition, the visual Schisto POC-CCA cassette score of the Schisto POC-CCA test was compared to the score of its respective standard Schisto POC-CCA test. The score was classified as below if it was below the standard, same if it was similar to the standard, and high if it was higher than the standard. Each DUS extraction condition’s performance was evaluated by looking at the proportion of their tests that scored below, similar, and higher than the standard.

### 2.4 Ethics statement

The study protocol was reviewed and approved by the Muhimbili University of Health and Allied Sciences Ethical Review Board (reference no. DA.282/298/01.C/1297). Permission to conduct the study in the Mwanza region was requested from the President’s Office - Regional Administration and Local Government, and the Regional Administrative Secretary of the Mwanza region. Specimens for the experiments were obtained from 14 anonymised volunteer fishermen at a fishing camp along Lake Victoria in the Mwanza region, as well as one volunteer project staff member who had no history of *S. mansoni* exposure. The participants were informed about the purpose of this study and gave their written consent. All participants were informed of their schistosomiasis testing results, and all *Schistosoma*-infected participants were offered free treatment (single 40mg/kg dose of Praziquantel).

## 3 Results

### 3.1 Schistosomiasis infection among fishermen

According to the formal-ether, Kato-Katz, and Schisto POC-CCA techniques, the number of *S. mansoni* positive fishermen were 7, 6, and 9, respectively. The average intensity of infection was 172 EPG, with a range of 24 to 528 EPG. Of the 6 positive fishermen by the Kato-Katz technique, 3 had light-intensity of infections, 2 had moderate-intensity of infections and 1 had heavy-intensity of infection. Out of 9 fishermen with Schisto POC-CCA positive results, 5 had 2+ and 4 had 3+ visual score results. There was no *Schistosoma haematobium* egg in any of the fishermen urine and volunteer project staff. Furthermore, all direct solvent tests yielded negative results.

### 3.2 Optimization of the number of punches and solvent volume

**Table 1** shows the results of each trial conducted to optimize the number of DUS punches and solvent volume. The results show that the trial using 5 punches in 150 µl of any of the three solvents produced a sufficient volume of eluate for Schisto POC-CCA cassette testing. Furthermore, the eluate colour from this trial (5 punches in 150 µl) strongly matched the original urine sample.

**Table 1:** Summarizes the results of optimization of DUS punches and solvent volume.

Trial no.	Number of punches	Volume of solvent (µl)	Resulted eluate volume (µl)	Visual (colour) resemblance with original urine sample
1^st^	3	120	< 100	Strong
2^nd^	3	135	≥ 100	Week
3^rd^	4	135	< 100	Strong
4^th^	4	150	≥ 100	Moderate
5^th^	5	150	≥ 100	Strong

### 3.3 Performance of optimized dried urine spot extraction conditions

**Figure 3:**
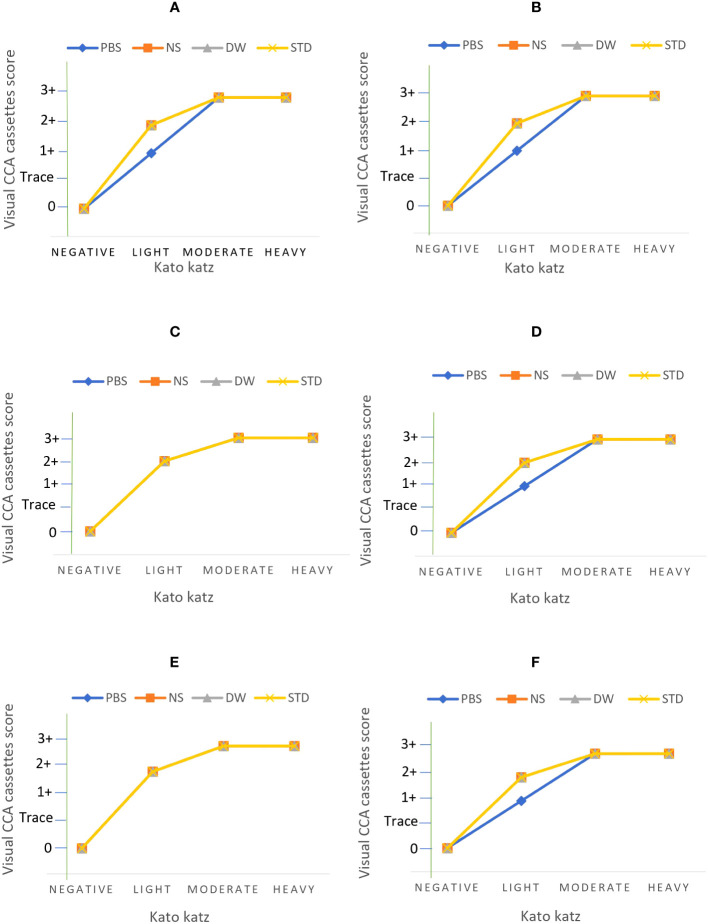
Results of assays performed on DUS samples 24 hours after preparation at the following extraction conditions; **(A)** 4 ^0^C incubation for 24 hours, **(B)** 4 ^0^C incubation for 2 hours, **(C)** room temperature incubation for 24 hours, **(D)** room temperature incubation for 2 hours, **(E)** 37 ^0^C incubation for 24 hours, and **(F)** 37 ^0^C incubation for 2 hours. Note: PBS stands for phosphate buffered saline, NS stands for normal saline, DW stands for distilled water, and STD stands for standard diagnostic test.

**Figure 4:**
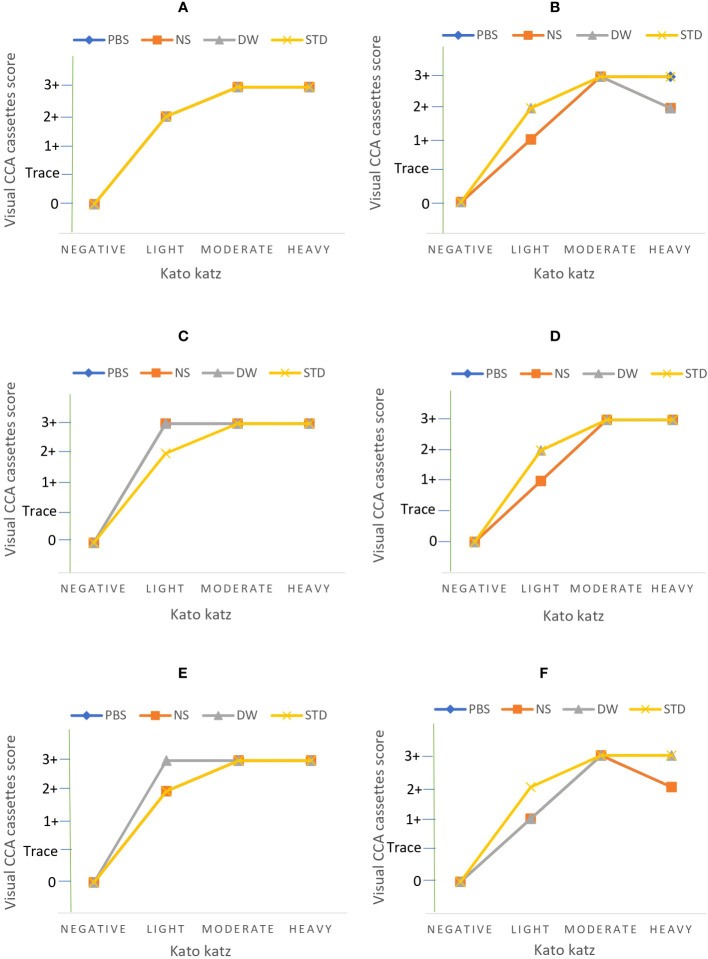
Results of assays performed on DUS samples 7 days after preparation at the following extraction conditions; **(A)** 4 ^0^C incubation for 24 hours, **(B)** 4 ^0^C incubation for 2 hours, **(C)** room temperature incubation for 24 hours, **(D)** room temperature incubation for 2 hours, **(E)** 37 ^0^C incubation for 24 hours, and **(F)** 37 ^0^C incubation for 2 hours. PBS stands for phosphate buffered saline, NS stands for normal saline, DW stands for distilled water, and STD stands for standard diagnostic test.

A total of 144 assays were run to evaluate different conditions for *S. mansoni* CCA extraction from DUS samples. These conditions included the type of extraction solvent, incubation time, and temperature (**Figure 2**). When 24-hour-old DUS specimens were incubated for 24 hours at room temperature or 37^0^ C, the same results as the standard were obtained for all solvents. Similar results were obtained when DUS samples dissolved in normal saline and distilled water were incubated for 2 hours at 4^0^ C, 2 hours at room temperature, 2 hours at 37^0^ C, and 24 hours at 4^0^ C. It was found that, when a DUS sample with Kato-Katz light intensity results was dissolved in phosphate buffered saline and incubated for 2 hours at 4^0^ C, 2 hours at room temperature, 2 hours at 37^0^ C, and 24 hours at 4^0^ C, it had poor CCA recovery when compared to the same urine sample tested using the standard direct urine test (**Figure 3**). When 7 days-old DUS specimens were incubated for 24 hours at any of the 3 temperatures, the same or better results than the standard were obtained for all solvents. The DUS (with Kato-Katz light intensity results) dissolved in normal saline incubated for 2 hours at 4^0^ C, room temperature, and 37^0^ C, and in distilled water and phosphate buffered saline incubated for 2 hours at 37 ^0^C, have lower CCA recovery when compared to the standard direct urine test. The DUS (with Kato-Katz heavy intensity results) dissolved in normal saline incubated for 2 hours at 4 ^0^C and 37^0^ C, dissolved in distilled water incubated for 2 hours at 4^0^ C, and dissolved in phosphate buffered saline incubated for 2 hours at 37 ^0^C all showed low recovery (**Figure 4**). Furthermore, all dried solvent spots yielded negative results for both 24 hours-old and 7 days-old samples.

In general, distilled water outperformed other solvents across all DUS assays when compared to the standard assays. A total of 47 (98.0%) out of 48 DUS assays that used distilled water produced the same or high performance than their respective standard assays, while 43 (89.6%) DUS assays that used normal saline and 40 (83.3%) DUS assays that used phosphate buffered saline produced the same or high performance than their respective standard assays. Furthermore, 2 (4.2%) DUS assays that used distilled water outperformed their respective standard assays, while only 1 (2.1%) DUS assay for each of normal saline and phosphate buffered saline outperformed their respective standard assays (**Table 2**).

**Table 2:** Shows the overall performance of DUS Schisto POC-CCA assays for each solvent when compared to their respective standard Schisto POC-CCA assays.

Solvent	Performance n (%)			Total number of tests
	Below	Same	Higher	
Phosphate buffered saline	8 (16.7)	39 (81.2)	1 (2.1)	48
Normal saline	5 (10.4)	42 (87.5)	1 (2.1)	48
Distilled water	1 (2.1)	45 (93.8)	2 (4.2)	48
Total	14 (9.7)	126 (87.5)	4 (2.8)	144

n, number of tests.

In terms of incubation temperature, the DUS assays performed better at room temperature than at other temperatures (4^0^ C and 37^0^ C). A total of 45 (93.7%) out of 48 DUS assays incubated at room temperature yielded the same or higher performance than their respective standard assays, while 43 (89.6%) DUS assays incubated at 4^0^ C and 42 (87.5%) DUS assays incubated at 37 ^0^C yielded the same or higher performance than their respective standard assays. Furthermore, 3 (6.2%) DUS assays incubated at room temperature outperformed their respective standard assays, while 1 (2.1%) DUS assay incubated at 37^0^ C outperformed its respective standard assay. No DUS assay had performed better than its respective standard assay when incubated at 4^0^ C (**Table 3**).

**Table 3:** Shows the overall performance of DUS Schisto POC-CCA assays for each incubation temperature condition when compared to their respective standard Schisto POC-CCA assays.

Temperature	Performance n (%)			Total number of tests
	Below	Same	Higher	
4 ^0^C	5 (10.4)	43 (89.6)	0 (0.0)	48
Room temperature	3 (6.2)	42 (87.5)	3 (6.2)	48
37 ^0^C	6 (12.5)	41 (85.4)	1 (2.1)	48
Total	14 (9.7)	126 (87.5(	4 (2.8)	144

n, number of tests.

Regarding incubation time, DUS assays incubated for 24 hours performed better than those incubated for 2 hours when both are compared to the standard assays. A total of 71 (98.7%) out of 72 DUS assays incubated for 24 hours yielded the same or higher performance than their respective standard tests, while 59 (81.9%) out of 72 DUS assays incubated for 2 hours yielded the same or higher performance as their respective standard assays. Furthermore, 4 (5.6%) DUS assays incubated for 24 hours outperformed their respective standard assays. No DUS assay had performed better than its respective standard assay when incubated for 2 hours (**Table 4**).

**Table 4:** Shows the overall performance of DUS Schisto POC-CCA assays for each incubation time condition when compared to their respective standard Schisto POC-CCA assays.

Incubation time	Performance n (%)			Total number of tests
	Below	Same	Higher	
2 hours	13 (18.1)	59 (81.9)	0 (0.0)	72
24 hours	1 (1.4)	67 (93.1)	4 (5.6)	72
Total	14 (9.7)	126 (87.5)	4 (2.8)	144

n, number of tests.

## 4 Discussion

DMS is a sample collection strategy that involves soaking and drying a small volume of a fluid, such as blood, urine, saliva or sweat, onto filter paper (19). DMS is increasingly being used to collect specimens for various purposes ranging from quantitative approaches like therapeutic drug monitoring to qualitative approaches like disease diagnosis or doping testing (19). The method has had a significant impact on several fields of study, including newborn screening, epidemiology (field testing), infectious diseases, environmental research, forensics, therapeutic drug monitoring, illicit drug analysis, toxicology, and toxico- and pharmacokinetic studies of drugs and candidate drugs (20). DUS is a DMS method that has recently gained a lot of attention. DUS sampling is a filter paper method that has been used to collect urine to test for by-products of adrenal and sex steroid hormones and their respective metabolic pathways, providing a gauge for understanding the body’s hormone metabolism (21, 22). Furthermore, DUS sampling has been used to test drugs (19), neurotransmitters, and elements such as iodine and metals such as arsenic (21, 22). Barry and colleagues were the first to develop procedures for DUS sampling method during procedures for the detection of phenylketonuria and the *Lactobacillus arabinosus* microbiological assay (23, 24).

The WHO recommends the use of Schisto POC-CCA for mapping of schistosomiasis of *Schistosoma mansoni* in endemic countries (25). This study developed methods for preparation of DUS on filter papers and detecting *Schistosoma* CCA in the DUS eluate using the Schisto POC-CCA cassette test. Many parameters influence assay performance and must be assessed and optimized, as in most assay development studies, to produce a standardized protocol that gives maximum recovery (maximum results) of the analyte of interest. The efficiency of the assay parameters must be assessed and optimized in order to produce a standardized protocol before an assay can be provisionally accepted and moved on to the validation stage (26). In this study, parameters assessment and optimization included trial and error experiments with extensive reagents (solvents) and filter paper measurements and evaluation of critical components (volume of urine to saturate on filter paper, volume of solvent, solvent type, and number of filter paper punches to dissolve) as well as assay conditions (incubation times and incubation temperatures), and result interpretation (definition of negative and positive results, further classification of positive results, and their meaning).

Several methods for preparing DUS have been tested and used. The methods include directly soaking filter paper in client urine. In this method, the client is instructed to saturate the provided filter paper with his or her urine by urinating directly on it. The other method is for a client to collect his or her urine in a container and then dip the provided filter paper in the urine. After saturating the filter paper, the client is instructed to hang it on a towel rack or other object to dry for 2 to 24 hours at room temperature without allowing the filter paper to touch anything. After drying, the client should place the filter paper in a ziploc bag with desiccant and bring it to the laboratory in person or by mail. These methods are commonly used for clients who require 24-hour urine collection and are typically performed at the client’s home (27, 28). The third method involves providing a client with a urine container in which the urine is collected and delivered to the laboratory or expert personnel in the field. The personnel then complete all of the remaining procedures for preparing DUS. Personnel may prepare the DUS in this procedure by either dipping the filter paper in the urine container or transferring (spotting) urine onto the filter paper using pipette (29, 30). In our study, we opted for the third method of DUS preparation; transferring (spotting) urine onto the filter paper by using pipette. This is because most of the clients in schistosomiasis studies are children or people with very low knowledge of handling urine as potentially hazardous specimen. In addition, the use of expert personnel is ideal because it ensure that appropriate volume of urine (5-6 drops for the case of our study) is saturated and evenly distributed on the required area of the filter paper as it is usually assumed that the analytes (including *Schistosoma* CCA) are distributed uniformly in the urine.

Analyte extraction is the most important stage of DMS (including DUS) sample preparation before analyte detection. Elution is the commonly used method for extracting analytes from DUS. Through this method, analytes are extracted from DUS by washing the spots with a suitable solvent (19, 31, 32). In this study, we also employed elution to extract *Schistosoma* CCA from DUS on filter paper. Because the amount of urine specimen spotted onto filter paper is limited, the elusion process is critical for obtaining sufficient analytes concentrations for accurate test results. Therefore, the analyte concentration in the eluate from a DUS must quantitatively reflect the original liquid urine sample concentration. Researchers have tried to identify things that must be considered during the elution process in order to achieve maximum dissociation of analyte from filter paper. The first things to consider are DUS size and solvent volume. Before using DUS specimens in a specific test, appropriate-sized pieces of filter paper specimens must be sampled by punching from the large DUS paper and dissolving them in an appropriate volume of solvent. To ensure that we achieve maximum extraction of *Schistosoma* CCA, we considered DUS size by optimizing the various number of DUS punches and dissolving them in different solvent volumes (**Table 1**). We assumed that by eluting DUS in such a way that the eluate resembled the original urine sample, most of the analytes (including *Schistosoma* CCA) would be dissociated from the filter paper in greater quantities. In this study, this assumption was met by dissolving five punches (6 mm diameter each) of DUS made on Whatman 3 filter paper in 150 µl of each of the three solvents (distilled water, phosphate buffered saline, and normal saline).

The other parameter that should be taken into consideration during DUS elution is the type of solvent used. The effects of different elution solvents on the DMS analytes concentrations in eluate have been extensively evaluated and need to be taken into consideration when developing a DMS method (33). For example, a urine sample may contain multiple excreted analytes; failing to adequately select the appropriate elution solvent may result in increased extraction of non-target analytes, increasing the possibility of cross-reactivity and, ultimately, a decreased ability to detect the analyte of interest by a specific diagnostic test method. Furthermore, some elution solvents may react and denature the analyte of interest, reducing its detection. We did not look into the chemical reactions that take place between the solvents we assessed and *Schistosoma* CCA. However, because the *Schistosoma* CCA is a glycoprotein, we attempted to use the most common solvents used for protein extraction (distilled water, phosphate buffered saline and normal saline). Our findings showed that, among these solvents, distilled water performed better than the other two solvents. When compared to phosphate buffered saline and normal saline, aqueous solutions are commonly used as protein analyte elution solvents. In general, an aqueous solvent is used for protein analytes extraction as it promotes analyte stability hence increasing its detection (34).

The temperature and time of elution are also important things to consider. Elution methods frequently require DUS to be dissolved in tubes containing the elution solution at specific temperature and time. Most procedures have method-specific incubation temperatures that range from 4 ^0^C to room temperature (25 ^0^C) for an hour to 24-hours (33). *Schistosoma* CCA is a very temperature-stable glycoprotein that is not easily distracted by minor temperature changes (35); however, temperature may have indirect effects by causing other analytes in DUS or elution solvents to affect the dissociation or nature of CCA and thus its concentration in the eluate. In our study, we included the core internal human body temperature (37 ^0^C) in addition to the two common temperatures (4 ^0^C and room temperature). However, after these temperatures were optimized, many assays incubated at room temperature performed better. In contrast, other studies found that 37 ^0^C was the optimal temperature for extracting urine analytes from DUS on filter papers (31, 32). In terms of incubation time, we discovered that incubating the elution solution for 24 hours yielded the best results when compared to 2 hours. The same results were reported in methods that used elution to extract urine analytes from DUS on filter papers (32).

## 5 Conclusion

This study provides proof of the feasibility of collecting urine samples (DUS) using filter paper and detecting *Schistosoma* CCA from DUS samples using the Schisto POC-CCA cassette test. Because DMS methods are cost-effective, the use of filter paper-based DUS for urine collection and detection of *Schistosoma* CCA will ease the schistosomiasis work for control programs and researchers in resource-limited settings by allowing them to test both recent samples for clinical care and older samples, regardless of transportation and storage issues.

## 6 Study limitations

One of the important limitations of this study was the lack of technique to actually quantify the concentration of *Schistosoma* CCA in the DUS eluates and their original urine samples. However, to mitigate this we carefully compared the visual appearance of the eluate with its original urine sample. Another constraint was the use of filter paper from a single manufacturer. The qualities of filter papers produced by various companies vary. The difference could be attributed to the materials used or the manufacturing process. The materials used to manufacture the filter paper were also suggested to have effects on the analytes in the DUS. For example, it has been proposed that hydroxyl and carboxylic groups on the hydrophilic surface of cellulose interact with the *Schistosoma* circulating anodic antigen, resulting in low antigen recovery. Moreover, filter paper from different manufacturers may have different pore sizes and fiber densities. These variations may influence the quality of the filter paper to maintain the *Schistosoma* CCA. Another limitation of the study is that it was a proof-of-concept study with limited sample sizes. To validate the results of this study, we recommend a larger study with a larger sample size to be conducted.

## Data availability statement

The original contributions presented in the study are included in the article/supplementary material. Further inquiries can be directed to the corresponding author.

## Ethics statement

The studies involving humans were approved by Muhimbili University of Health and Allied Sciences Ethical Review Board. The studies were conducted in accordance with the local legislation and institutional requirements. The participants provided their written informed consent to participate in this study.

## Author contributions

AZ conceptualized the study. AZ, TM, CK and FL designed the study. AZ, CK and GO participated in data acquisition. AZ analysed and interpreted the data. AZ, TM drafted the manuscript. BN revised the work critically for important intellectual content. All authors contributed to the article and approved the submitted version.
